# Ultrasensitive visual read-out of nucleic acids using electrocatalytic fluid displacement

**DOI:** 10.1038/ncomms7978

**Published:** 2015-04-22

**Authors:** Justin D. Besant, Jagotamoy Das, Ian B. Burgess, Wenhan Liu, Edward H. Sargent, Shana O. Kelley

**Affiliations:** 1 Institute for Biomaterials and Biomedical Engineering, University of Toronto, Toronto, Canada M5S 3G9; 2 Department of Pharmaceutical Science, Leslie Dan Faculty of Pharmacy, University of Toronto, Toronto, Canada M5S 3M2; 3 Department of Electrical and Computer Engineering, Faculty of Engineering, University of Toronto, Toronto, Canada M5S 3G4; 4 Department of Biochemistry, Faculty of Medicine, University of Toronto, Toronto, Canada M5S 1A8

## Abstract

Diagnosis of disease outside of sophisticated laboratories urgently requires low-cost, user-friendly devices. Disposable, instrument-free testing devices are used for home and physician office testing, but are limited in applicability to a small class of highly abundant analytes. Direct, unambiguous visual read-out is an ideal way to deliver a result on a disposable device; however, existing strategies that deliver appropriate sensitivity produce only subtle colour changes. Here we report a new approach, which we term electrocatalytic fluid displacement, where a molecular binding event is transduced into an electrochemical current, which drives the electrodeposition of a metal catalyst. The catalyst promotes bubble formation that displaces a fluid to reveal a high contrast change. We couple the read-out system to a nanostructured microelectrode and demonstrate direct visual detection of 100 fM DNA in 10 min. This represents the lowest limit of detection of nucleic acids reported using high contrast visual read-out.

## Abstract (web summary)

Point-of-care analytical devices are of interest for diagnostic applications where larger scale laboratory instruments are not feasible or available. Here, the authors present a direct read-out colorimetric sensor which uses catalytic gas production to visualize picomolar concentrations of DNA.

## Graphical abstract

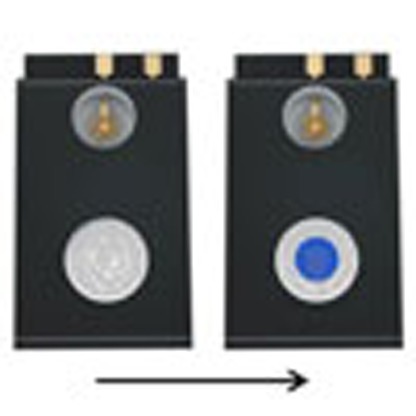

Low-cost, user-friendly diagnostics have the potential to expand the ubiquity of molecular testing in clinical medicine^1^,^2^,^3^,^4^,^5^,^6^,^7^,^8^,^9^,^10^,^11^. Disposable, instrument-free devices are used today, but have so far only achieved the detection of certain analytes that happen to be highly abundant. A key feature of these devices is the use of an easy-to-interpret visual read-out strategy. Existing read-out approaches require the accumulation of a high level of an analyte, and therefore only abundant analytes have been detected visually. Developing ways to link a visible, unambiguous colour change to rare biological molecules remains an unmet need. Recently, a variety of direct, colorimetric read-out strategies have been reported: these include approaches based on nanoparticles^12^,^13^, plasmonic nanomaterials^14^, 2D materials^15^ and enzymatic reactions^7^. Unfortunately, these approaches require interpretation of subtle colour changes. This can make analyses operator-dependent, or, in other cases, diminishes the benefits of a test being instrument-free by requiring a scanner device.

Developing new, easy-to-interpret interfaces that convey diagnostic results obtained with low-abundance analytes would enable the development of low-cost diagnostics for a spectrum of new diseases. Motivating this work are rapid recent advances in biosensors that produce nanoampere electrical current changes as a function of specific biomarkers present in a sample^16^,^17^,^18^. New strategies to transduce extremely small electrochemical currents into easily perceived, high-contrast visual changes would allow the visual detection of low abundance analytes using electrochemical biosensors. In addition, low-cost current-to-colour conversion is of broad interest in displays and in sensors for non-medical applications.

Strategies for direct colorimetric read-out of electric currents include paper-based electrochromism^19^, electrochromic polymers^20^, metal oxides^21^ and fluorescent dyes^22^. Electrochromic polymers and dyes allow for rapid and reversible colour switching in response to electrical currents, but the currents required to switch areas detectable to the naked eye are above the threshold necessary for sensitive electrochemical detection. Inducing visible colour changes using currents below 1 μA is a fundamental challenge, for such currents fail to supply enough electrons to electrochemically reduce a visibly perceptible quantity of electrochromic material. Directly translating such low currents into visible changes has yet to be achieved without the aid of costly, power-consumptive active electronics such as amplifiers.

We here develop an approach to amplify the changes to optical density triggered by the levels of electrochemical current generated at a nucleic acid sensor. We term our new approach electrocatalytic fluid displacement (EFD). An electrochemical current drives the deposition of a catalyst, which promotes the growth of a bubble that actuates a fluid. Specifically, the electrochemical current drives the electrodeposition of a metal catalyst for hydrogen peroxide decomposition. On the introduction of hydrogen peroxide liquid, a bubble catalytically forms, and this displaces a fluid. The bubble displaces a dye, or, in the alternative, modifies the index of refraction to reveal a structural colour change. We begin by providing a conceptual basis for our approach, and we benchmark it against other colorimetric read-out strategies. After optimizing the device parameters and geometry, we determine the minimum current necessary for successful colorimetric read-out. To showcase this approach, we demonstrate sensitive colorimetric detection of ssDNA by coupling the read-out to a nanostructured microelectrode (NME) and a novel electrocatalytic assay.

## Results

### Overview of electrocatalytic fluid displacement

**Figure 1:**
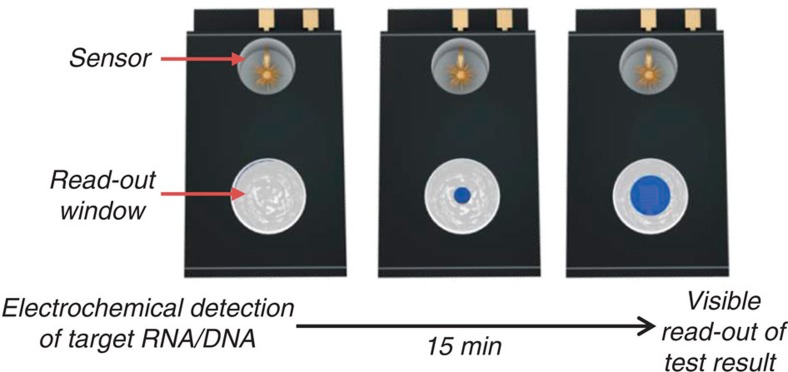
Schematic of an integrated device for electrocatalytic fluid displacement. An electrochemical current from a nanostructured microelectrode is converted into a visible change through the deposition of a catalyst that catalyses bubble formation. As the bubble grows, the white dye is displaced to reveal a blue colour.

**Figure 2:**
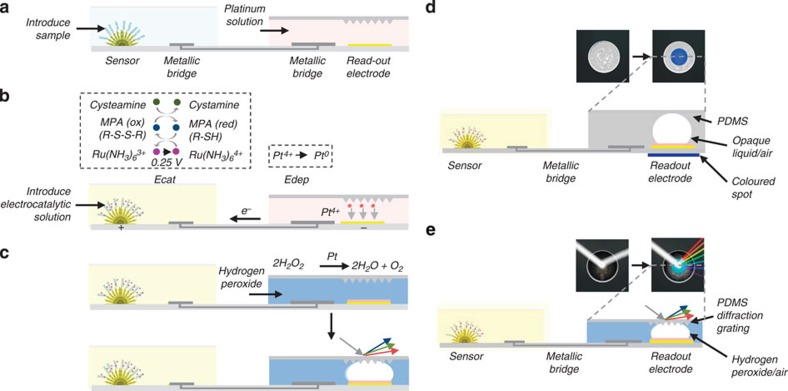
Colorimetric detection of DNA using electrocatalytic fluid displacement. (**a**) Target hybridization. The analyte hybridizes to a complementary PNA probe. Ru(NH_3_)_6_^3+^ is electrostatically attracted to the negatively charged backbone of the target nucleic acid. (**b**) Signal transduction. A potential is applied to the NME which oxidizes Ru(NH_3_)_6_^3+^. The current is amplified using an electrochemical-chemical-chemical (ECC) reporter system. Ru(NH_3_)_6_^3+^ is regenerated by MPA, which is in turn regenerated by cysteamine. The electrochemical current drives the deposition of platinum, a catalyst for hydrogen peroxide decomposition, on a mesh electrode immersed in platinum solution. (**c**) Colorimetric read-out. After the introduction of peroxide, a bubble forms as the platinum catalyses the decomposition of peroxide. The growing bubble is transduced into a colour change either through an optical density change or a structural colour change. In the optical density approach, the bubble displaces a white dye to reveal the blue spot. To induce a structural colour change, the bubble displaces peroxide that causes an index mismatch at a diffraction grating patterned in the underside of the chamber lid. Incident white light is diffracted into its component colours. (**d**) Optical density change. A cross-section of the electrocatalytic fluid displacement approach with read-out based on dye displacement. (**e**) Structural colour change. A cross-section of the electrocatalytic fluid displacement approach with read-out based on a structural colour change. In the case of electrocatalytic fluid displacement based on a structural colour change, the underside of the PDMS lid of the read-out chamber is patterned with a diffraction grating.

The electrocatalytic fluid displacement (EFD) approach is based on the electrodeposition of platinum, which catalyses the evolution of a bubble that actuates a fluid (Fig. 1). An electrochemical sensor is connected to a read-out chamber by a metallic bridge (Fig. 2a). On the introduction of the sample, the target analyte hybridizes to the complementary probe functionalized on the surface of the sensor (Fig. 2a). After hybridization, the electrocatalytic solution is introduced into the sensing chamber (Fig. 2b). On the application of a potential at 250 mV for 10 s, ruthenium is oxidized at the sensing electrode and platinum is simultaneously electrodeposited at a mesh electrode in the read-out chamber. The current is further amplified by two additional reducing agents in the electrocatalytic solution. After the application of the potential, hydrogen peroxide is introduced into the read-out chamber (Fig. 2c). The deposited platinum catalyses the decomposition of hydrogen peroxide into water and oxygen, which forms a bubble. The growing bubble displaces a fluid to reveal a colour change. In the case of read-out based on dye displacement, the opaque dye is displaced and the read-out window becomes transparent revealing a blue spot underneath the device (Fig. 2d). In the case of read-out based on a structural colour change, the growing bubble causes an index mismatch that unveils the diffraction grating patterned in the underside of the PDMS chamber lid, which causes light to diffract into its component colours (Fig. 2e).

To sense nucleic acids, the EFD read-out system is connected to a nanostructured microelectrode (NME), which acts as an ultrasensitive electrochemical biosensor (Fig. 2a)^17^,^23^. The NME sensors were fabricated on silicon substrates using a two-step electrodeposition process as previously described^23^. The gold microstructures protrude from the surface and reach into solution, which increases the probability of interaction with the target molecules^23^. The sensors are decorated with a second layer of finely nanostructured gold. The nanoscale roughness maximizes sensitivity by enhancing the hybridization efficiency of the probe and target^24^,^25^. These sensors have been used previously to detect a variety of chemical and biomolecular targets^26^,^27^,^28^.

We use a multi-pronged strategy to minimize the current in the absence of target nucleic acid. We functionalize the sensors using a charge-neutral probe, and we read the current using a novel electrochemical assay. Specifically, the sensors are functionalized with thiolated peptide nucleic acid (PNA) probes complementary to the target sequence. PNA is a synthetic nucleic acid analogue that has a neutral charge. This neutral charge minimizes the background current and increases the signal-to-noise ratio.

After target hybridization and washing (Fig. 2a), we read-out the electrochemical signals using an electrochemical-chemical-chemical (ECC) redox cycle reporter system, which radically amplifies the current. It is worth noting that this is the first reported use of ECC for the detection nucleic acids^29^. To simplify the electronics in a disposable device, we use a DC potential for read-out as opposed to voltammetry, which requires a potential sweep and thus more complicated electronics. Using a DC potential, it is not possible to resolve the contribution from unwanted redox reactions occurring at nearby potentials to the overall signal. In the past we have used an electrocatalytic redox reporter system consisting of ruthenium hexamine and ferricyanide for nucleic acid detection using differential pulse voltammetry^30^, but this gave high background currents using DC potential amperometry. At the reduction potential of ruthenium hexamine, ferricyanide is reduced as well, which contributes to the overall current even in the absence of bound-target nucleic acids. Therefore, we designed the ECC redox reporter system such that there are no interfering redox reactions near the potential of interest.

Our new assay employs Ru(NH_3_)_6_^3+^, mercaptopropionic acid (MPA) and cysteamine. Ru(NH_3_)_6_^3+^ is electrostatically attracted to the negatively charged phosphate backbone of the bound target nucleic acids. On the application of a potential at 250 mV, Ru(NH_3_)_6_^3+^ is oxidized to Ru(NH_3_)_6_^4+^ (ref. 31). The MPA present in solution chemically reduces Ru(NH_3_)_6_^4+^ back to Ru(NH_3_)_6_^3+^, allowing for multiple turnovers of Ru(NH_3_)_6_^3+^, which generates a high electrocatalytic current. This signal is further amplified by cysteamine, another reducing agent, which is chemically oxidized to cystamine by reducing the oxidized form of MPA (R-S-S-R) back to its reduced form (R-SH).

This electrical current from the sensor is coupled to the EFD electrode immersed in a platinum solution to drive the deposition of the catalyst. When the NME is challenged with the target analyte, the current drives the electrodeposition of platinum on the EFD electrode, which catalytically forms a bubble that displaces the dye to reveal the blue spot. When the target sequence is not present, the current is too low to deposit a sufficient amount of platinum to catalyse bubble growth and no colour change occurs.

### A comparative model of colour change

Catalytic electrochromic transduction methods offer significant signal amplification needed for transducing the ultra-low currents generated by the ECC assay compared with direct electrochromic reduction. To study the prospective performance of this approach, we calculated the predicted time required to induce a visible colour change using a variety of transduction strategies.

We illustrate the challenge of directly inducing a colour change by considering the example of electrodepositing an optically discernible quantity of metal. A 1 nA current applied for 10 s supplies 6 × 10^10^ electrons, which can turnover a maximum of 6 × 10^10^ molecules. Even under the generous assumption that a single molecular layer is visible, given an atomic radius of 1 Å, this yields a spot of only 50 μm × 50 μm. This is too small to be easily visible to the naked eye as the spatial resolution of human eyesight is ∼100–200 μm (ref. 32).

**Figure 3:**
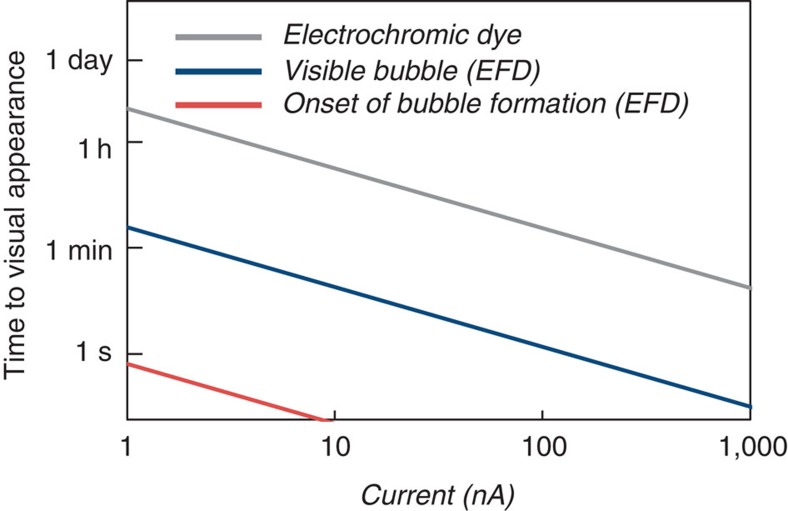
Calculations of the time to visual appearance. We calculated the time to visual appearance using electrocatalytic fluid displacement and reduction of an electrochromic compound. We assume the read-out window is a 200 μm × 200 μm × 50 μm chamber and the current is applied for 10 s. The onset of bubble formation occurs as the solution is saturated with oxygen. A bubble is defined as visible once it reaches the volume of the chamber. We assumed the electrochromic dye had the absorbance of malachite green and a visible change corresponds to a ΔOD of 1.

We hypothesized that we could instead develop a means to amplify, by orders of magnitude, the colour change per charge. We would electrodeposit a catalyst, such as platinum, to turn on the colorimetric reaction^19^. By depositing a catalyst, each electron effectively converts multiple molecules, amplifying the colour transformation. However, as Fig. 3 shows, even the catalytic reduction of an electrochromic compound in bulk solution requires exceedingly long times to induce a visible change. Assuming a 50-μm tall chamber with a 200 μm diameter window filled with enough pigment, with the absorbance of malachite green, to give an OD of 1, it would take over 4 h to turnover the compound using the platinum deposited from a 1 nA current.

Thus, instead of catalytic reduction of a solution-based pigment, we considered catalytic evolution of gas as an equivalent molar amount of gas occupies a much larger volume than a liquid. At STP, the volume of one mole of gas is 22.4 l, which is three orders of magnitude larger than a mole of liquid H_2_O (18 ml). Platinum is an excellent catalyst for the decomposition of hydrogen peroxide to form oxygen and water^33^. As shown in Fig. 3, the catalytic production of a visible bubble that fills the same window requires under 3 min, over 80 times faster than catalytic reduction of an electrochromic dye in solution.

We hypothesized that electrocatalytic bubble formation could be converted into a colorimetric change by actuating a fluid to modulate the optical density (OD) of the read-out window. This is a central step in EFD.

### Optimization of device geometry

**Figure 4:**
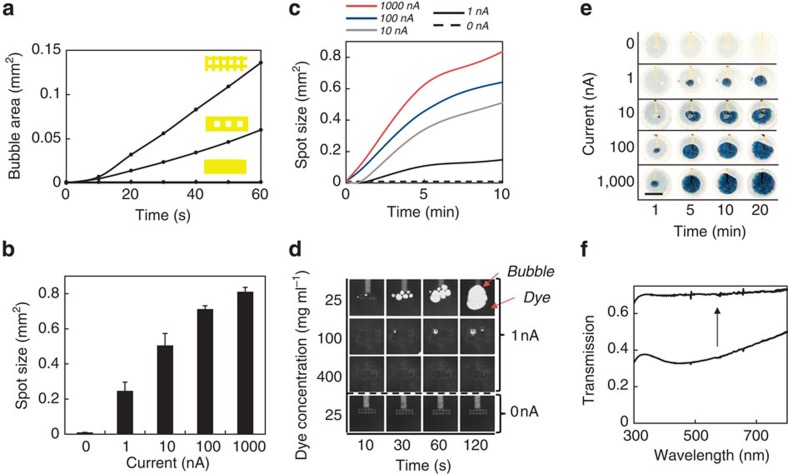
Electrocatalytic fluid dye displacement. (**a**) Bubble evolution as a function of time for various electrode geometries. Platinum was deposited using a 1 nA current for 10 s. Bubble growth increases with the ratio of edges to surface area. (**b**) Average bubble area after 20 min as a function of applied current using the electrodes with the highest mesh density. The bubble is confined to a 50-μm tall channel. Error bars represent s.e.; all measurements represent n>5 trials. (**c**) Bubble growth as a function of time for various deposition currents using electrodes with the highest mesh density. Bubbles do not form when no current is applied. (**d**) Images of bubble growth as a function of dye concentration acquired using an optical microscope. (**e**) Images of colorimetric read-out as a function of deposition current and time. One-nA currents are detectable in 5 min. Scale bar, 1 mm. (**f**) Transmission spectrum of the read-out window before and after bubble growth.

Motivated by our calculations, we sought to experimentally validate the electrocatalytic fluidic displacement approach. We patterned a rectangular gold electrode on a glass substrate, which sits at the bottom of a 50-μm tall by 1.5-mm wide circular chamber. After depositing platinum for 10 s at 1 nA, we introduced hydrogen peroxide and measured the rate of bubble growth using a microscope (Fig. 4a). Although we did not observe rapid bubble growth, we noticed that bubbles formed preferentially at the electrode edges.

To test the enhancement provided by edges, we designed mesh-shaped electrodes with increased ratios of edges to surface area. We applied 1 nA for 10 s to deposit platinum and recorded the rate of bubble growth (Fig. 4a). We found that the rate of bubble evolution increased with increasing numbers of edges. The highest density mesh, with 3.4 × the edge to surface area ratio of the rectangular electrode, provided the fastest bubble growth. No bubbles formed when no current was applied as no platinum was electrodeposited. Bubble growth was not observed after immersing the device in platinum solution for 25 min, indicating that platinum is not deposited via electroless deposition (Supplementary Fig. 1).

Using the high-density mesh electrodes, we measured the average growth of the bubble for various applied currents. Figure 4b shows the average bubble area measured after 20 min as a function of electrodeposition current while Fig. 4c shows the bubble growth over time. After 20 min, a 1 nA current applied for 10 s yields a bubble with an area of 0.25 mm^2^, which is visibly detectable.

### Electrocatalytic fluidic dye displacement

To induce a visible colour change that is easily interpretable by the end-user, we utilize the bubble to displace an opaque dye that obscures a blue spot beneath the read-out window. As the chamber fills with oxygen, the blue spot is revealed.

Increasing the dye concentration increases the opacity of the dye, but also increases its viscosity. We found that at higher viscosities, bubble formation was inhibited (Fig. 4d). We optimized the dye concentration and found that using a concentration of 25 μg ml^−1^ allowed for sufficient optical density to conceal the blue spot while promoting bubble growth (Fig. 4d).

To determine the minimum visibly detectable current, we deposited platinum at various rates for 10 s and measured the exposed area of the blue spot (Fig. 4e). Using a 1 nA deposition current, the spot area grows to 0.09 mm^2^ in 5 min. The exposed area expands to 0.24 mm^2^ by 20 min. No bubble growth is observed when platinum is not electrodeposited. As the spatial resolution of human eyesight is about 200 μm (ref. 32) the smallest visible area ∼200 μm × 200 μm or 0.04 mm^2^. Thus, the spot area of 0.09 mm^2^ obtained from a 1 nA current after 5 min is visible to the naked eye.

To quantify the performance of our device we calculated the colouration efficiency, a metric that quantifies the efficiency of converting an electrical current into a colorimetric change. Colouration efficiency, CE, is given by:


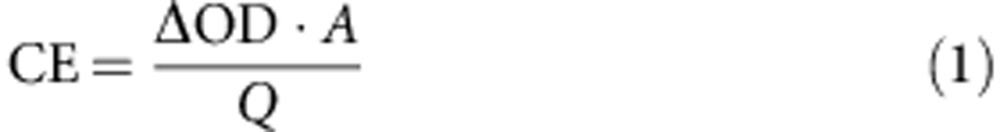


**Figure 5:**
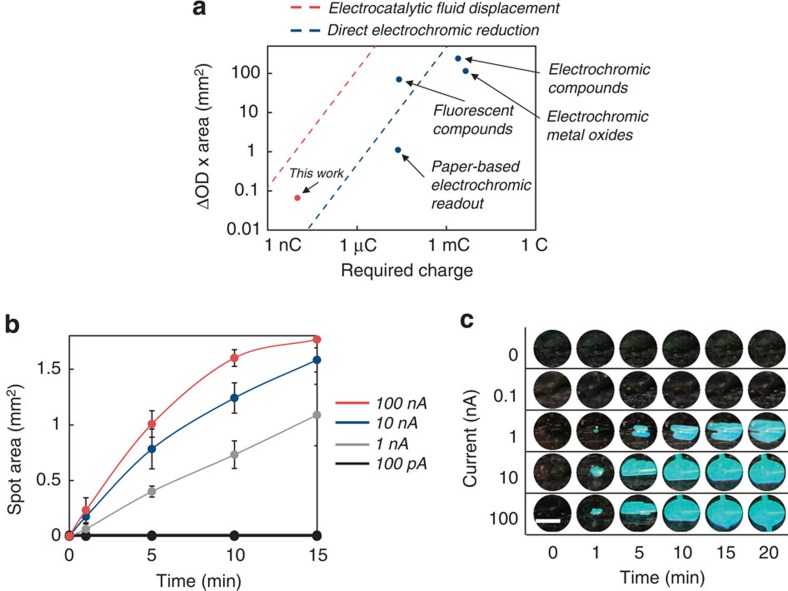
Electrocatalytic fluid displacement reveals a structural colour change. (**a**) Comparison of the charge required to induce a visible colour of a certain area and optical density change for a variety of read-out strategies. The dashed red line represents the calculated exposed area of a bubble generated using electrocatalytic fluid displacement. We assume the bubble is confined to a 50-μm tall chamber, the reaction proceeds for 10 min, and the ΔOD is 1. The dashed blue line represents the area of a monoatomic layer of platinum directly reducible by the current. We assume the ΔOD is 1 and thus this represents an upper bound using this strategy. (**b**) Spot size as a function of time for various deposition currents using electrodes with the highest mesh density. Bubbles do not form when no current is applied. Error bars represent s.e. (*n*=3). (**c**) Images of colorimetric read-out as a function of deposition current and time using a diffraction grating. The window turns from optically transparent (which appears as black due to a black background) to cyan as light at that wavelength is diffracted towards the camera. One-nA currents are detectable in 1 min. Scale bar, 1 mm.

Where ΔOD is the change in optical density, *Q* is the charge required for switching [C] and *A* is the spot area [cm^2^]. We measured the optical density before and after switching and found a ΔOD of 0.27 (Fig. 4f). Given a switchable area of 0.24 mm^2^ after 20 min using a 1 nA current applied for 10 s, this device has a colouration efficiency of 6.48 × 10^4^ cm^2^ C^−1^. Figure 5a compares the switchable area as a function of charge for devices with the highest reported colouration efficiencies for a range of read-out strategies. Given the previous records of 2.6 × 10^4^ cm^2^ C^−1^ for fluorescent polymers^22^ and 9.3 × 10^2^ cm^2^ C^−1^ for non-fluorescent electrochromic compounds^20^, a colouration efficiency of 6.48 × 10^4^ cm^2^ C^−1^ is, to our knowledge, the highest reported value in the literature for an electrochromic device.

### Induction of a structural colour change

As optical absorbance increases with path length, the read-out window must be sufficiently tall for the dye to obscure the coloured spot beneath. This limits the response time of a colorimetric device based on dye displacement, as the bubble must grow large enough to reach the chamber ceiling.

By patterning substrates with feature sizes on the order of the wavelength of light, it is possible to produce vibrant structural colours^34^. Examples of this include diffraction gratings and iridescence. The colour of the substrate can be modified by matching the index refraction between a second medium and the substrate^35^.

We hypothesized that we could exploit a structural colour change to decrease the read-out turnaround time. As structural colour changes rely on the index matching at an interface, the colour change is largely independent of the path length through the index-matching medium. Thus, we could expect a vibrant colour change using a device with a much smaller channel height than required when using dye displacement. As the substrate provides the colour, there is no need to increase the opacity of the peroxide by introducing additional compounds, which might interfere with the reaction.

To prove out this approach, we patterned a diffraction grating into the underside of the PDMS lid affixed to the top of the device with a 7-μm tall channel. As the index of refraction of peroxide (*n*=1.35) is similar to that of PDMS (*n*=1.4), the diffraction grating is invisible to incoming light when the device is initially loaded with peroxide. As the bubble forms, the peroxide is replaced with O_2_, which has an index of refraction of 1. This index mismatch between the bubble and PDMS unveils the diffraction grating. The incident white light is diffracted into its component colours to reveal the circular spot.

Figure 5b shows the growth of the coloured spot using the diffraction grating approach while Fig. 5c shows the corresponding images of the spot over time. As the bubble grows, white light begins to diffract into its component colours. The window turns from optically transparent (which appears as black due to a black background) to cyan as light at that wavelength is diffracted towards the camera. Using a 1 nA deposition current, the spot size is 0.06 mm^2^ after 1 min, which is visible by eye. This spot grows to 0.36 mm^2^ and 1.1 mm^2^ by 5 and 15 min, respectively. Given a spot size of only 0.1 mm^2^ after 5 min using electrocatalytic fluidic displacement of a dye, the structural colour spot of 0.36 mm^2^ is over three times larger in the same time frame. No spot forms when no current is applied Fig. 5c.

### Colorimetric read-out of ssDNA

**Figure 6:**
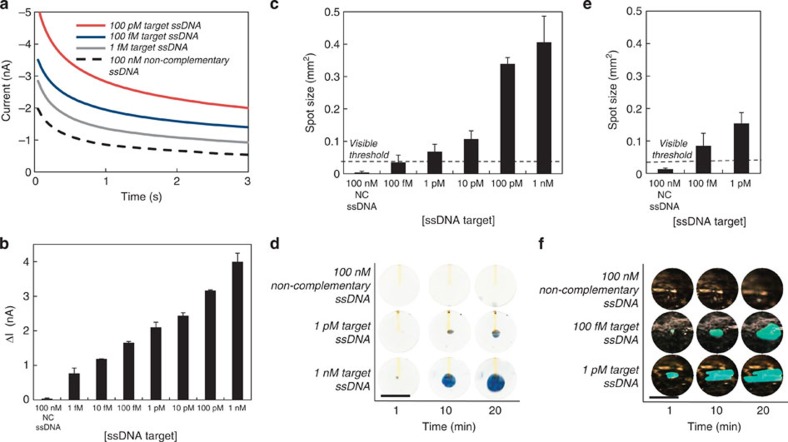
Colorimetric detection of DNA. (**a**) Electrochemical current as a function of time for various analyte concentrations after applying 250 mV w.r.t. to a Ag/AgCl reference electrode for 3 s. (**b**) Average peak electrochemical current as a function of analyte concentration. Negative control is non-complementary (NC) DNA. (**c**) Spot size as a function of target DNA concentration after 10 min using dye displacement. One-pM ssDNA is detectable by eye. The visible threshold is defined as an area of 200 mm × 200 mm. (**d**) Images of the EFD device showing growth of the bubble over time as a function of ssDNA concentration using dye displacement. (**e**) Spot size as a function of target DNA concentration after 10 min using a structural colour change. (**f**) Images of the EFD device showing growth of the bubble over time as a function of ssDNA concentration using a structural colour change. Scale bar, 1 mm. Error bars represent s.e.; all measurements represent *n*>5 trials.

To test the capability of the EFD device to detect biomarkers, we connected the NME sensors in serial (Supplementary Fig. 2) to the EFD read-out chip and challenged the NMEs with ssDNA. As an initial characterization of the ECC assay, we challenged the sensors with serial dilutions ssDNA. We then measured the corresponding currents after applying 250 mV (Fig. 6a). The average peak current decreases with decreasing target ssDNA concentration giving a detection limit of 1 fM (Fig. 6b). The current generated from 100 nM non-complementary ssDNA is less than 2 nA, which is similar to the background current, indicating this read-out method is specific.

To demonstrate colorimetric read-out of biomarkers, we coupled the assay to our read-out device and challenged the sensors with serial dilutions of ssDNA. To connect the sensors to our EFD device, we immersed the NME sensors in the ECC solution and the EFD read-out device in the platinum electrodeposition solution. To bridge electronically the sensor and read-out device, a platinum wire electrode immersed in the ECC solution is connected to a second platinum electrode in the electrodeposition bath. The EFD read-out device acts as the counter electrode for the entire system (Fig. 2). After applying 250 mV for 10 s to the NME, we introduced peroxide into the EFD chip and measured the rate of colour formation (Fig. 6c). We found a detection limit of 1 pM after 10 min with an average spot size of 0.068 mm^2^. To our knowledge, a detection limit of 1 pM is the lowest reported limit of detection for colorimetric detection of ssDNA using an electrochemical sensor. No visible spot was observed when the sensors were challenged with 100 nM of non-complementary ssDNA indicating a specificity discrimination ratio of 1 × 10^5^ (Fig. 6d).

We studied the performance of the diffraction grating approach for colorimetric ssDNA detection. First, we optimized the peroxide concentration to minimize bubble formation from currents at the background level. We found that bubble growth at low currents could be suppressed using 10% peroxide (Supplementary Fig. 3). We challenged the devices with ssDNA and measured the growth of the diffracting area (Fig. 6e). Figure 6f shows the corresponding images of the growth of the visible spot over time. Using 1 pM complementary ssDNA, the spot size was 0.15 mm^2^ after 10 min. In that same time frame, the spot using dye displacement was 0.068 mm^2^, which is about two times smaller. This is expected as the chamber height using the diffraction grating approach was five times smaller than the chamber in the dye displacement device, and thus the bubble can rapidly grow laterally. Using this method, 100 fM of ssDNA was also detectable by eye with an average spot size of 0.085 mm^2^ (Fig. 6e). No spot was visible with 100 nM non-complementary ssDNA (Fig. 6f).

## Discussion

This colorimetric read-out approach is an inexpensive, disposable and low-power alternative to using electronics for read-out of electrochemical currents. We use three stages of amplification to transform ultra-low currents into colorimetric changes. The ECC redox assay uses two chemical catalysis steps to amplify the electrodeposition current. Thus, each bound nucleic acid is converted into multiple deposited platinum atoms. Next, by electrodepositing a catalyst, the colorimetric reaction continues long after the initial application of the electrochemical current, obviating the need for high currents to induce colorimetric changes. Last, colorimetric read-out is accelerated by exploiting the fact that a gas occupies a much larger volume than an equivalent molar amount of liquid. A catalytic reaction can evolve a large volume of gas much faster than it can turnover a visible amount of dye of the same volume.

Even though spot sizes as small as 100–200 μm are visible under perfect conditions to those with 20/20 vision, spot sizes <1 mm^2^ may be difficult to see for some. Small spot sizes could be easily magnified using inexpensive lenses fabricated from elastomers such as PDMS.

A fully integrated device would require the timed introduction of reagents with automated flow. This could be integrated onto an instrument-free device using passive fluidic systems such as paper microfluidics, capillary pumps or on-chip vacuum pumps^3^,^36^,^37^. As this strategy only requires the application of a DC potential, the potential could be applied using a DC power source such as a battery as opposed to a potentiostat.

In summary, we introduce a strategy for rapid and sensitive colorimetric read-out of electrochemical currents based on electrocatalytic fluid displacement. This approach relies on the electrochemical mediated deposition of platinum which catalyses the growth of a fluid displacing bubble. We present two strategies for converting this fluidic displacement into a visible colour change using a dye and a structural colour change. We demonstrate successful colorimetric detection of a 1 nA current in 1 min and calculate a colouration efficiency of 6.48 × 10^4^ C cm^−2^, which to our knowledge is the highest value reported in the literature. We showcase this approach by coupling our device to a novel electrocatalytic assay and nanostructured microelectrode sensor to demonstrate successful and specific colorimetric detection of 100 fM of ssDNA in 10 min with a discrimination ratio of over 1 × 10^5^.

## Methods

### Calculation of time required for visible bubble formation

To calculate the rate of colour change using direct electrochromic colorimetric read-out, we assume a channel 50-μm tall by 200-μm wide filled with enough electrochromic dye to give an OD of 1. We assumed a high molar absorptivity of 1 × 10^7^ M^−1^ m^−1^, which is similar to that of malachite green. Using the catalysis rate of platinum, we calculate the time needed to turn over the dye in the channel. To calculate the rate of colour change using electrocatalytic fluidics, we assume a channel a chamber that is 50-μm tall with a 200-μm width. Using the catalysis rate of platinum, we calculated the rate of oxygen formation. The onset of bubble formation occurs as peroxide in the chamber is saturated with oxygen. We assume the bubble is visible once it grows to the volume of the chamber. Parameters used in the calculations are listed in Supplementary Table 1.

### Device fabrication

The device was fabricated using standard photolithographic methods. In brief, electrodes were patterned on a glass substrate. The device was passivated using SU-8 2002 (Microchem, Newton, MA) and apertures were patterned to expose the electrodes below. The channel was fabricated by patterning SU-8 3050 (Microchem, Newton, MA).

### Platinum electrodeposition

The electrode was immersed in K_2_PtCl_4_ (Sigma-Aldrich, MO) and connected to an Epsilon potentiostat (BASi West Lafayette, IN) using a three-electrode set-up with a Ag/AgCl reference electrode and a Pt counter electrode. Using chronopotentiometry, various currents were applied for 10 s. After electrodeposition, the device was washed thoroughly with H_2_O and covered with a PDMS (Dow Chemical, MI) lid.

### Colorimetric read-out using a dye

Hundred microlitres of white dye (Liquitex Titanium White Ink) was centrifuged for 5 min at 15 000 *g*. The supernatant was removed and replaced with 400 μl of 30% H_2_O_2_ (Sigma-Aldrich, MO). The dye (25 μg ml^−1^) was introduced into the channel and the amount of bubble generation was measured over time using a camera (Canon).

### Colorimetric read-out using structural colour

A diffraction grating was patterned in PDMS by curing PDMS on a DVD-R. The PDMS diffraction grating lid was removed and attached to the device with a 7-μm tall channel patterned using SU-8 2010 (Microchem, Newton, MA). Twenty-seven percent H_2_O_2_ with 1% pluoronic (Sigma-Aldrich, MO) was introduced into the device, and colour changes were measured over time using a camera (Canon).

### Sensor chip fabrication

Six-inch silicon wafers (University Wafer, MA) were passivated using a thick layer of thermally grown silicon dioxide and coated with a 25 nm Ti adhesion layer. A 350-nm gold layer was deposited on the chip using electron-beam-assisted gold evaporation, which was again coated with 5 nm of Ti. The electrodes were patterned in the metal layers using standard photolithography and a lift-off process. A 500 nm layer of insulating Si_3_N_4_ was deposited using chemical vapour deposition. The 5-μm apertures were etched at the tips of the metal leads using standard photolithography. Contact pads (0.4 mm × 2 mm contact) were patterned using wet etching as well.

### Fabrication of sensors

Chips were cleaned by sonication in acetone for 5 min, rinsed with isopropyl alcohol and DI water, and dried with nitrogen. Electrodeposition was performed at room temperature. The 5-μm apertures on the fabricated electrodes were used as the working electrodes and were contacted using the exposed contact pads. Nanostructured microelectrode sensors were electrodeposited in a solution of 50 mM HAuCl_4_ (Sigma-Aldrich, MO) and 0.5 M HCl (Sigma-Aldrich, MO) using DC potential amperometry at 0 mV for 100 s. After washing with DI water and drying, the sensors were coated again with a thin layer of Au to form nanostructures by plating at−450 mV for 10 s.

### Functionalization of sensors

An aqueous solution containing 1 μM of probe (5′-GGT CAG ATC GTT GGT GGA GT-3′) (PNA Bio, CA) was mixed with 10 μM of aqueous Tris(2-carboxyethyl)phosphine hydrochloride solution (Sigma-Aldrich, MO) and then the mixture was left for overnight to cleave disulphide bonds. After mixing 100 nM of 6-mercaptohexanol (MCH) (Sigma-Aldrich, MO) to this probe solution mixture, 20 μl was pipetted onto the chips and incubated for 3 h in a dark humidity chamber at room temperature for probe immobilization. The chips were then washed thrice for 5 min with 0.1 × PBS (Life Technologies, CA) at room temperature. The chips were then treated with 1 mM MCH for an hour at room temperature for back filling. After washing, the chips were challenged with different concentration of targets for 30 min at room temperature. After hybridization, the chips were washed thrice for 5 min with 0.1 × PBS at room temperature and the electrochemical scans were acquired.

### Electrochemical detection of ssDNA

All electrochemical experiments were carried out using a Bioanalytical Systems Epsilon potentiostat with a three-electrode system featuring a Ag/AgCl reference electrode and a platinum wire auxiliary electrode. Electrochemical signals were measured in a Tris buffer solution (50 mM, pH 9) containing 10 μM [Ru(NH_3_)_6_]Cl_3_ (Sigma-Aldrich, MO), 0.5 mM 3-mercaptopropionoic acid (MPA) (Sigma-Aldrich, MO) and 0.5 mM cysteamine (Cys) (Sigma-Aldrich, MO). DC potential amperometry (DCPA) signals were obtained at +250 mV for 10 s. Signal changes, Δ*I*, were calculated with Δ*I*=*I*_c_–*I*_0_ (where *I*_c_ is the current at a given concentration and *I*_0_ is the current without analyte).

## Author contributions

J.D.B, J.D, I.B.B., W.L., E.H.S. and S.O.K. designed the experiments. J.D.B, J.D, I.B.B. and W.L. performed the experiments and interpreted results with assistance from E.H.S. and S.O.K.; J.D.B., J.D., I.B.B., W.L., E.H.S. and S.O.K. composed and refined the manuscript.

## Additional information

**How to cite this article:** Besant, J.D. *et al*. Ultrasensitive visual read-out of nucleic acids using electrocatalytic fluid displacement. *Nat. Commun*. 6:6978 doi: 10.1038/ncomms7978 (2015).

## Supplementary Material

Supplementary Information Supplementary Figures 1-3, Supplementary Table 1
