# Supplementary material for: Ultrasensitive visual read-out of nucleic acids using electrocatalytic fluid displacement
Source: Nat Commun. 2015 Apr 22;6:6978. doi: 10.1038/ncomms7978 (PMC4421844; doi:10.1038/ncomms7978)
Supplement: Supplementary Information — Supplementary Figures 1-3, Supplementary Table 1 [file ncomms7978-s1.pdf]

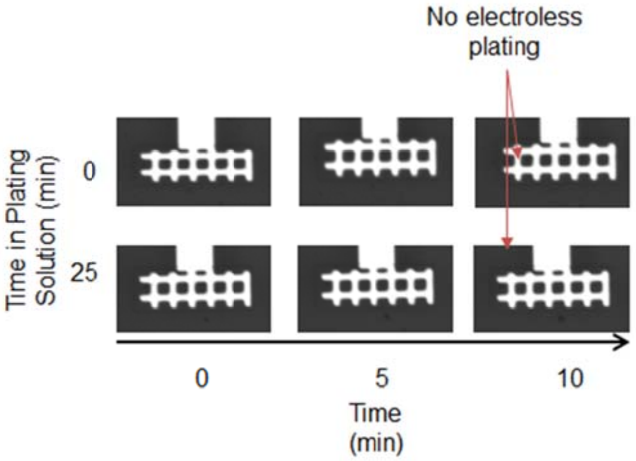

Supplementary Figure 1 | Effect of electroless deposition. Electrodes were immersed in 30% H_2O_2 before and after dipping in a platinum solution for 25 minutes (no potential was applied). Bubbles do not form after 10 minutes in either case, indicating there is no appreciable electroless deposition.

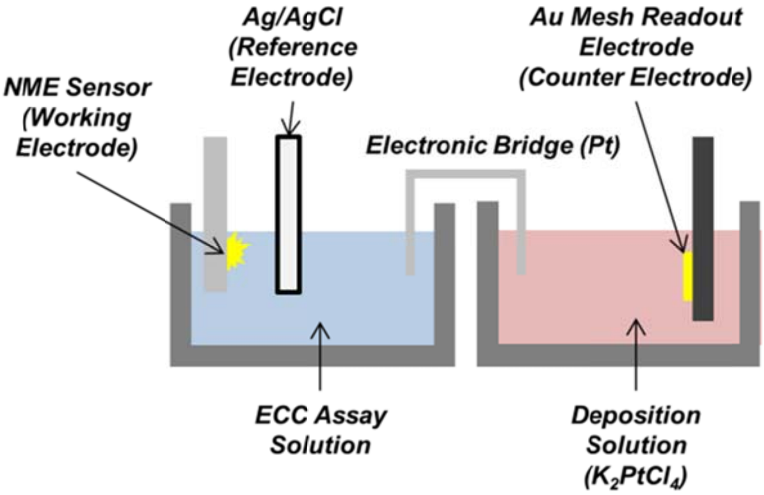

Supplementary Figure 2 | Setup used for electrochemical sensing. The NME acts as the working electrode and the Au mesh readout electrode acts as the counter electrode. A platinum wire serves as an electronic bridge between the two solutions.

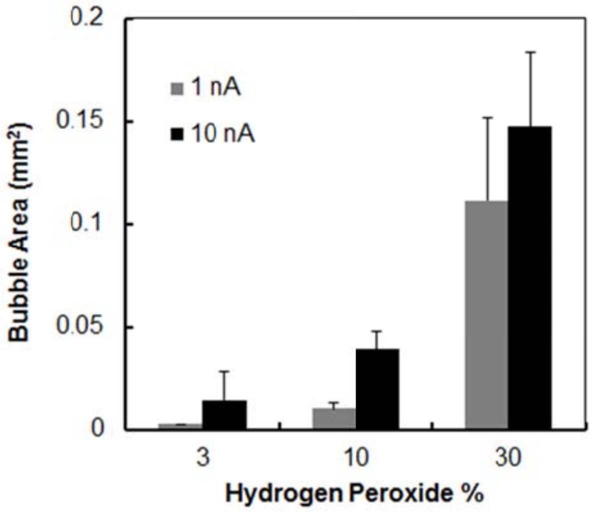

Supplementary Figure 3 | Effect of hydrogen peroxide concentration on bubble growth. We measured the effect of hydrogen peroxide concentration on bubble growth after 2 minutes in peroxide solution for various applied currents. By tuning the peroxide concentration it is possible to control the rate of bubble growth. When 3% peroxide is used, no bubbles form after applying a 1 nA deposition current.

Description	Value
Channel Height	50 µm
Channel Width	200 µm
Solubility of Oxygen in Water	7.6 mg/L
Catalysis rate of platinum	8.84×10^{-4} mol s^{-1} m^{-2}
Hydrogen Peroxide Concentration	15%
Molar absoprtivity of the electrochromic dye	1×10^{7} M^{-1} m^{-1}
Optical Density (OD) of the Dye in Channel	1

Supplementary Table 1 | Parameters used to calculate time to visual appearance. Parameters used in calculating the time required to induce a visible change using various methods.
